# In-Hospital and long term results of primary angioplasty and medical therapy in nonagenarian patients with acute myocardial infarction

**DOI:** 10.15171/jcvtr.2017.25

**Published:** 2017-09-30

**Authors:** Muslum Sahin, Lutfi Ocal, Ali Kemal Kalkan, Alev Kilicgedik, Mehmet Emin Kalkan, Burak Teymen, Ugur Arslantas, Mehmet Muhsin Turkmen

**Affiliations:** ^1^Kartal Kosuyolu Heart Education and Research Hospital, Department of Cardiology, Istanbul, Turkey; ^2^Mehmet Akif Ersoy Thoracic and Cardiovascular Surgery Training and Research Hospital, Department of Cardiology, Istanbul, Turkey; ^3^Emsey Hospital, Department of Cardiology, Istanbul, Turkey

**Keywords:** Nonagenarian, Elderly, Myocardial Infarction, Primary Percutaneous Intervention

## Abstract

***Introduction:*** Although percutaneous coronary intervention is an accepted "first-line" therapy in acute ST elevation myocardial infarction (STEMI) in general population, few data are available on the short- and long-term outcomes of very old patients (age >90 years). Our aim is to evaluate and compare the short and long-term outcomes after primary percutaneous coronary intervention (PPCI) or medical therapy in nonagenarian patients with STEMI.

***Methods:*** We retrospectively identified patients older than 90 years old in our clinic, with acute STEMI who presented within 12 hours after symptoms onset, either underwent PPCI or medically treated. In hospital events and long-term results analyzed subsequently.

***Results:*** From January 2005 to December 2014, 73 patients with STEMI either underwent PPCI (PPCI group n = 42) or had only medical treatment (Non-PPCI group n = 31). Mean age was 92.4 ± 3.1 (90-106). Patients were followed 26.5 ± 20.1 months. Recurrent myocardial infarction during hospitalization was not observed in both groups. In-hospital mortality, cerebrovascular events and acute renal failure rate were similar between two groups (respectively *P * = 0.797 and *P * = 1.000, *P * = 0.288), whereas arrhythmia was significantly higher in the PPCI group ( 0; 21.4%, *P * = 0.009). Results show re-infarction was similar in both groups (respectively 3.2%; 11.9%, *P * = 0.382) but mortality in long-term was significantly lower in the PPCI group (respectively 40.9%; 12.9%, *P* = 0.02).

***Conclusion:*** In nonagenarian patients, with STEMI mortality is very high. Although; in-hospital events were similar, the long-term mortality rate was significantly lower in patients treated with PPCI.

## Introduction

The elderly patient population is rapidly increasing in the developed countries. Scientists are expecting that until 2020 there will be 2.6 million nonagenarians (≥90 age) in the United States.^1^ The prevalence of coronary artery disease increases with age.^2^ Also previous studies demonstrated that there is high mortality and morbidity risk in patients with acute myocardial infarction with advancing age.^3-5^ Percutaneous coronary intervention is an accepted treatment form in patients with STEMI^6^ due to the latest guidelines. But the management of older (>90 years old) nonagenarian patients with STEMI is still controversial. Therefore we aimed to evaluate and compare the in-hospital and long-term outcomes in nonagenarian’s patients with STEMI either treated with primary percutaneous coronary intervention (PPCI) or medical therapy.

## Materials and Methods

From January 2005 to December 2014; 73 STEMI patients within 12 hours after symptoms onset and aged ≥90 years are retrospectively identified. The inclusion criteria were admission within the first 12 hours of the onset of typical ischemic chest pain and ST elevation of at least 1 mm in two or more contiguous leads (2 mm for V1–V3) or new-onset left bundle branch block (LBBB). In the recent STEMI guidelines, LBBB is no longer STEMI equivalent. But patients who had new-onset LBBB were included in this study due to the study period was between 2005-2014.

The benefits and risks of the reperfusion therapy (PPCI and thrombolytic therapy) have been told to the all patients and patients’ relatives. 42 patients accepted PPCI, 4 patients accepted thrombolytic therapy but 31 patients rejected any reperfusion therapy due to concern about the risk of intracerebral hemorrhage. Patients that received thrombolytic therapy were excluded from the study due to a small number of patients. In addition the patients experiencing more than 12 hours of pain, and patients with non-STEMI and allergy to any of the drugs used (aspirin, clopidogrel, GP IIb/IIIa inhibitors) were excluded from the study.

Demographic measures, risk factors, and comorbidities are summarized in Table 1. The primary endpoints of this study are, in hospital and long-term outcomes. The patients were divided into two groups: PPCI group (n = 42) and non-PPCI group (medical group: with antiischemic, antiplatelet, anticoagulant therapy and clinical follow-up) (n = 31). All patients were given aspirin 300 mg and clopidogrel 300-600 mg on admission in the emergency department. PPCI was performed by femoral access using 6-7 F guiding catheter in all patients. Intravenous unfractioned heparin 100 IU/kg was administered to achieve an activated clotting time of >250 seconds. at the beginning of the procedure. Additional heparin, guided by activat­ed clotting time, was administered dur­ing percutaneous coronary intervention (PCI). Thrombus aspiration catheter and glycoprotein IIb/IIIa inhibitors were used accordingly to the physician’s preference in each individual patient. Also either bare metal stent or drug eluting stent were implanted due to operator’s discretion. A successful procedure was defined as infarcted artery stenosis <30% and Thrombolysis In Myocardial Infarction (TIMI) grade 2 or 3 flow. PPCI was not performed in non-PPCI group. In the non-PPCI group, the patients were admitted to CCU and their electrocardiograms were continuously monitored for at least 72 hours. Hemodynamic monitoring by an arterial line and pulmonary artery balloon catheters was performed in patients with advanced heart failure. Patients of non-PPCI group not received any reperfusion therapy. Heparin was routinely administered to each patient in this group for 3-5 days unless there was contraindication. Patients received 75 mg/d clopidogrel and 300 mg/d aspirin in both groups. All other medications, including , β–blockers, angiotensin-converting enzyme inhibitors, statins, nitrates, vasopressor agents, digoxin, vasodilator agents, antiarrhythmic drugs, calcium channel blockers, analgesics (morphine and meperidine), antiemetic drugs and diuretics were used as needed in both groups.

**Table 1:** Demographic and clinical data of groups

	**Non-PPCI group (n = 31**)	**PPCI group (n = 42)**	***P***
Age	92.3±4.0	91.2±2.4	0.397
Sex (male)	9	10	0.788
Hypertension	19 (61.3%)	26 (61.9%)	1.000
Diabetes mellitus	14 (45.2%)	16 (38.1%)	0.633
Hyperlipidemia	3 (9.7%)	6 (14.3%)	0.724
Smoking	2 (6.5%)	5 (11.9%)	0.691
Chronic renal failure	16 (51.6%)	14 (33.%3)	0.151
Prior myocardial infarction	7 (22.6%)	5 (11.9%)	0.339
PCI history	3 (9.7%)	6 (14.3%)	0.724
By-pass history	0	0	
History of peripheral artery disease	4 (12.9%)	5 (11.9%)	1.000
Location of the infarction (anterior/non-anterior)	13/18	22/20	0.478
Systolic blood pressure (mm Hg)	123.7±28.3	115.0±29.7	0.200
Diastolic blood pressure (mm Hg)	72.9±16.8	62.2±16.7	0.009
Heart rate (bpm)	99.1±22.7	77.2±22.4	0.001
LVEF %	41.7±10.2	40.9±10.2	0.789
Valvular heart disease	17 (54.8%)	19 (45.2%)	0.191
Admission rhythm (sinus/non-sinus rhythm rate )	18 (58.1%)/13 (41.9%)	28 (66.7%)/14 (33.3%)	0.08
Length of hospitalisation (day)	6.1±4.7	7.7±8.7	0.400

Abbreviations: PCI, percutaneous coronary intervention; LVEF, left ventricular ejection fraction.

All participants underwent transthoracic echocardiography. Standard 2-dimensional echocardiographic examination was performed. Left ventricular ejection fraction (LVEF) was estimated by the modified Simpson rule.

In hospital outcome evaluated by death, re-infarction, cerebrovascular accident (CVA), severe arrhythmia, severe bleeding and acute renal failure. After hospitalization, all surviving patients were contacted by clinical interview or telephone. The patients were followed 26.5 ± 20.1 months and death, CVA, re-infarction were assessed during the follow-up. Recurrent myocardial infarction was defined as ischemic type chest pain associated with an increase in creatine kinase and creatine kinase- MB more than twice the last value and/or new ST segment elevation or new pathological Q waves. CVA was defined as a permanent loss of neurological function caused by an ischemic or hemorrhagic vascular event. Severe arrhythmia was defined as patients with supraventricular /ventricular tachycardia, atrial fibrillation and mobitz type 2/3 AV block. Acute renal failure was defined as an increase of 0.5 mg/dL in creatinine. Major bleeding was defined as required transfusion of >2 units of blood and/or the occurrence of intracranial/retroperitoneal hemorrhage.

### Statistical analysis

The data was analyzed using the SPSS software for Windows version 15.0 (SPSS Inc., Chicago, Illinois, USA). Continuous variables were expressed as mean ± standard deviation while categorical variables were expressed as percentage. Comparison of continuous values between two groups was performed by means of independent- samples *t* test. Categorical variables were compared by the chi-square test. A *P* value of <0.05 was considered statistically significant.

## Results

From January 2005 to December 2014**,** 73 patients with STEMI, either underwent PPCI (PPCI group n = 42) or had only medical treatment (Non-PPCI group n = 31). Mean age was 92.4 ± 3.1 (90-106). Nineteen patients were male and 54 were female. Patients were followed 26.5 ± 20.1 months. Demographic and clinical data were similar between the groups except the diastolic blood pressure and heart rate measurement on admission (diastolic blood pressure 72.9 ± 16.8 and 62.2 ± 16.7 mm Hg, *P* = 0.009; heart rate 99.1 ± 22.7 and 77.2 ± 22.4 bpm, *P* = 0.001). Infarct related artery was LAD in 22 patients and non-LAD in 20 patients in PPCI group. In 34 patients (bare metal stent n=18; drug eluting stent [DES] n = 16) we deployed stent, where in 8 patients we only performed PTCA. The average stent deployment per patient was 1.3 ± 0.6. The mean stent length was 28.9 ± 15.5 mm, whereas the mean stent diameter was 3.0 ± 0.4 mm. No-reflow phenomenon occurred in two patients. Both patients responded to intra coronary nitrate and adenosine administration. The technical success rate was 97.7% where one patient transferred to non-PPCI group.

LVEF and presence of severe valve disease were similar in both groups (respectively, *P* = 0.789; *P* = 0.191). Medical treatment after admission was similar in both groups only except glycoprotein IIb/IIIa treatment (1 patient in non-PPCI group, 4 patients in the PPCI group). Eight patients needed blood transfusion (3 patients in non-PPCI group, 5 patients in the PPCI group), but life-threatening bleeding was not observed in both groups. In-hospital mortality, cerebrovascular event, recurrent MI and acute renal failure rates were similar between the groups (respectively *P* = 0.797 and *P* = 1.000, *P* = 0.288). The rate of in-hospital severe arrhythmia was significantly higher in the PPCI group (0; 21.4%, *P* = 0.009) . The rate of acute MI was non-significantly higher in the PPCI group during the follow-up (respectively 3.2%; 11.9%, *P* = 0.382). All-cause mortality rate was significantly lower in the PPCI group (respectively 40.9%; 12.9%, *P* = 0.02) (Table 2).

**Table 2:** Short and long term results

	**Non-PPCI group (n = 31**) **No. (%)**	**PPCI group (n = 42)** **No. (%)**	***P***
In hospital recurrent MI	0 (0)	0 (0)	
In hospital death	9 (29)	11 (26)	0.797
In hospital cerebro-vascular event	0 (0)	1 (2.4)	1.000
In hospital stent thrombosis		0 (0)	
In hospital arrhythmia	0 (0)	9 (21.4)	0.009
In hospital decompensate congestive heart failure	5 (16)	8 (19)	1.000
In hospital acute renal failure	11 (35)	9 (21.4)	0.288
Long term (non-hospital) recurrent MI	1 (3.2)	5 (11.9)	0.382
Long term (non-hospital) death	9 (40.9)	4 (12.9)	0.02

Abbreviation: MI: myocardial infarction.

**Figure 1:**
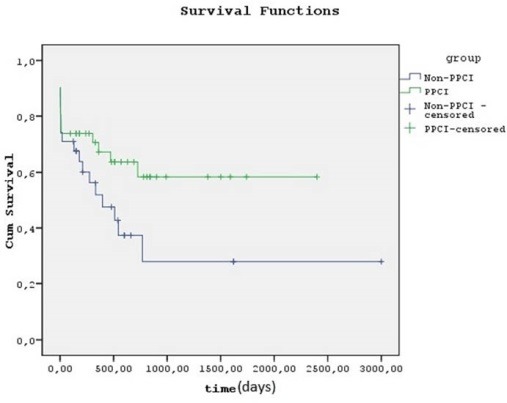
Kaplan-Meier survival curve in PPCI group and non-PPCI group patients .

Kaplan-Meier analysis demonstrated that PPCI group patients had a higher cumulative survival rate than non-PPCI group patients. But the difference was not statistically significant. (PPCI group, %64.3; Non-PPCI group, 41.9%; *P* = 0.085 by the log-rank test) (Figure 1).

## Discussion

Although older patients constitute a fast growing portion of cardiovascular patients, few data are available on the outcome of patients with STEMI undergoing primary angioplasty. To our knowledge, our study is the first trial to compare the short- and long-outcomes of PPCI versus only medical therapy without intervention.

Aortoiliac tortuosity is higher therefore coronary engagement may be difficult in older patients. Also frequency of complications is higher. Due to these reasons radial access or long sheath can be used in these patient cohort Instead of radial approach femoral approach was preferred in our study.

Previous studies demonstrated that high mortality and morbidity rates are associated with advancing age.^3^ The high mortality in this patient group linked to the presence of additional diseases. Shah et al suggested an in hospital mortality rate of 32% in nonagenarian patients with STEMI.^7^ Salinas et al reported that in hospital mortality rate of 34.2% in 38 nonagenarian patient with STEMI treated with PPCI.^2^ In another study in hospital mortality rate was 16%.^8^ Our results are similar compared to previous studies where in hospital mortality was 26% in PPCI, 29% in non-PPCI group. In >80 years, although shown to improve mortality rates with reperfusion therapy,^9^ there are limited data to guide STEMI therapy in nonagenarian patients. Costin et al suggested a survival benefit with PPCI compared to medical group in a small-populated study.^10^ In our study, in-hospital mortality was similar between two groups but in long-term follow-up mortality rate was significantly lower in the PPCI group.

Comorbid diseases are accompanied by more frequent in elderly patients.^1^ In our study, the most common cardiac risk factors were hypertension, diabetes mellitus, chronic renal failure and valvular heart disease. Conservative approach (medical therapy) has been the main treatment since very elderly patients will be accompanied by more frequent comorbid disease and there are more likely to occur of additional complications after any intervention.^2^ In elderly patients, previous studies showed that hemorrhage rates are higher in any form of reperfusion therapy.^11-13^ The risk of contrast-induced nephropathy is also expected to be higher in this patient cohort.^14,15^ In our study blood transfusion was needed in 8 patients, but life-threatening bleeding was not observed. Also femoral access path related complications did not occur. Our results suggested that dual anti-platelet therapy was safe in this patient population. Acute renal failure rate was high (27.3%), but it was similar in both groups. Based on these results, PPCI does not increase the risk of bleeding and acute renal failure development.

## Limitations

Limitations of the data presented include the retrospective, single-center design of the analysis, inadequate number of patients to allow statistically significant differences to be detected between the two groups.

## Conclusion

PPCI compared to conservative medical therapy reduces mortality in long-term follow-up in older patients (>90 years old) with STEMI.

## Ethical approval

The study complies with the principles outlined in the Declaration of Helsinki. The study was approved by our hos­pital Ethics Committee. Informed consent form is taken before all interventions in our institute and are being kept in the archives. Moreover all patients or patients’ relatives were contacted and informed about our study via phone call.

## Competing interests

The authors declare there is no conflict of interest.

## References

[1] Ionescu CN, Amuchastegui M, Ionescu S, Marcu CB, Donohue T (2010). Treatment and outcomes of nonagenarians with ST-elevation myocardial infarction. J Invasive Cardiol.

[2] Salinas P, Galeote G, Martin-Reyes R, Perez-Vizcayno MJ, Hernandez-Antolin R, Mainar V (2011). Primary percutaneous coronary intervention for ST-segment elevation acute myocardial infarction in nonagenarian patients: results from a Spanish multicentre registry. EuroIntervention.

[3] Hovanesyan A, Rich MW (2008). Outcomes of acute myocardial infarction in nonagenarians. Am J Cardiol.

[4] Kasanuki H, Honda T, Haze K, Sumiyoshi T, Horie T, Yagi M (2005). A large-scale prospective cohort study on the current status of therapeutic modalities for acute myocardial infarction in Japan: rationale and initial results of the HIJAMI Registry. Am Heart J.

[5] 
Carro
 
A
, 
Kaski
 
JC
 (2011). Myocardial Infarction in the Elderly. Aging Dis.

[6] Windecker S, Kolh P, Alfonso F, Collet JP, Cremer J, Falk V (2014). 2014 ESC/EACTS guidelines on myocardial revascularization: the Task Force on Myocardial Revascularization of the European Society of Cardiology (ESC) and the European Association for Cardio-Thoracic Surgery (EACTS) Developed with the special contribution of the European Association of Percutaneous Cardiovascular Interventions (EAPCI). Eur heart J.

[7] Shah P, Najafi AH, Panza JA, Cooper HA (2009). Outcomes and quality of life in patients greater than or equal to 85 years of age with ST-elevation myocardial infarction. Am J Cardiol.

[8] Valente S, Lazzeri C, Salvadori C, Chiostri M, Giglioli C, Poli S (2008). Effectiveness and safety of routine primary angioplasty in patients aged > or =85 years with acute myocardial infarction. Circ J.

[9] Alexander KP, Newby LK, Armstrong PW, Cannon CP, Gibler WB, Rich MW (2007). Acute coronary care in the elderly, part II: ST-segment-elevation myocardial infarction: a scientific statement for healthcare professionals from the American Heart Association Council on Clinical Cardiology: in collaboration with the Society of Geriatric Cardiology. Circulation.

[10] Ionescu CN, Amuchastegui M, Ionescu S, Marcu CB, Donohue T (2010). Treatment and outcomes of nonagenarians with ST-elevation myocardial infarction. J Invasive Cardiol.

[11] Antonsen L, Jensen LO, Thayssen P, Christiansen EH, Junker A, Tilsted HH (2011). Comparison of outcomes of patients >/=80 years of age having percutaneous coronary intervention according to presentation (stable vs unstable angina pectoris/non-ST-segment elevation myocardial infarction vs ST-segment elevation myocardial infarction). Am J Cardiol.

[12] Yi GY, Zhang XG, Zhang J, Wang X (2014). Factors related to the use of reperfusion strategies in elderly patients with acute myocardial infarction. J Cardiothorac Surg.

[13] Kinnaird TD, Stabile E, Mintz GS, Lee CW, Canos DA, Gevorkian N (2003). Incidence, predictors, and prognostic implications of bleeding and blood transfusion following percutaneous coronary interventions. Am J Cardiol.

[14] Rynkowska-Kidawa M, Zielińska M, Chiżyński K, Kidawa M. In-hospital outcomes and mortality in octogenarians after percutaneous coronary intervention. Kardiol Pol 2015 Jan 7. 10.5603/KP.a2014.0247 25563471

[15] Liu Y, Gao L, Xue Q, Yan M, Chen P, Wang Y (2014). Impact of renal dysfunction on long-term outcomes of elderly patients with acute coronary syndrome: a longitudinal, prospective observational study. BMC Nephrol.

